# Novel variation and *de novo* mutation rates in population-wide *de novo* assembled Danish trios

**DOI:** 10.1038/ncomms6969

**Published:** 2015-01-19

**Authors:** Søren Besenbacher, Siyang Liu, José M. G. Izarzugaza, Jakob Grove, Kirstine Belling, Jette Bork-Jensen, Shujia Huang, Thomas D. Als, Shengting Li, Rachita Yadav, Arcadio Rubio-García, Francesco Lescai, Ditte Demontis, Junhua Rao, Weijian Ye, Thomas Mailund, Rune M. Friborg, Christian N. S. Pedersen, Ruiqi Xu, Jihua Sun, Hao Liu, Ou Wang, Xiaofang Cheng, David Flores, Emil Rydza, Kristoffer Rapacki, John Damm Sørensen, Piotr Chmura, David Westergaard, Piotr Dworzynski, Thorkild I. A. Sørensen, Ole Lund, Torben Hansen, Xun Xu, Ning Li, Lars Bolund, Oluf Pedersen, Hans Eiberg, Anders Krogh, Anders D. Børglum, Søren Brunak, Karsten Kristiansen, Mikkel H. Schierup, Jun Wang, Ramneek Gupta, Palle Villesen, Simon Rasmussen

**Affiliations:** 1 Bioinformatics Research Center, Aarhus University, C. F. Møllers Allé 8, DK-8000 Aarhus, Denmark; 2 BGI Europe, Ole Maaløes Vej 3, DK-2200 Copenhagen, Denmark; 3 Department of Biology, University of Copenhagen, Ole Maaløes Vej 5, DK-2200 Copenhagen, Denmark; 4 Center for Biological Sequence Analysis, Department of Systems Biology, Technical University of Denmark, Kemitorvet 208, DK-2800 Kgs Lyngby, Denmark; 5 Centre for Integrative Sequencing, iSEQ, Aarhus University, Bartholins Allé 6, building 1242, DK-8000 Aarhus, Denmark; 6 The Lundbeck Foundation Initiative for Integrative Psychiatric Research, iPSYCH, DK-8000 Aarhus, Denmark; 7 Department of Biomedicine, Aarhus University, Bartholins Allé 6, building 1242, DK-8000 Aarhus, Denmark; 8 The Novo Nordisk Foundation Center for Basic Metabolic Research, Faculty of Health and Medical Sciences, University of Copenhagen, Universitetsparken 1–3, DK-2100 Copenhagen, Denmark; 9 School of Bioscience and Biotechnology, South China University of Technology, Guangzhou 510006, China; 10 Institute of Preventive Medicine, Bispebjerg and Frederiksberg Hospitals, The Capital Region, Nordre Fasanvej 57, Hovedvejen 5, DK2000 Copenhagen, Denmark; 11 Faculty of Health Sciences, University of Southern Denmark, DK-5000 Odense, Denmark; 12 Department of Cellular and Molecular Medicine, Panum Institute, University of Copenhagen, Blegdamsvej 3, DK-2200 Copenhagen, Denmark; 13 Centre for GeoGenetics, Natural History Museum of Denmark, University of Copenhagen, Øster Voldgade 5–7, DK-1350 Copenhagen, Denmark

## Abstract

Building a population-specific catalogue of single nucleotide variants (SNVs), indels and structural variants (SVs) with frequencies, termed a national pan-genome, is critical for further advancing clinical and public health genetics in large cohorts. Here we report a Danish pan-genome obtained from sequencing 10 trios to high depth (50 × ). We report 536k novel SNVs and 283k novel short indels from mapping approaches and develop a population-wide *de novo* assembly approach to identify 132k novel indels larger than 10 nucleotides with low false discovery rates. We identify a higher proportion of indels and SVs than previous efforts showing the merits of high coverage and *de novo* assembly approaches. In addition, we use trio information to identify *de novo* mutations and use a probabilistic method to provide direct estimates of 1.27e−8 and 1.5e−9 per nucleotide per generation for SNVs and indels, respectively.

## Abstract (web summary)

The generation of a national pan-genome, a population-specific catalogue of genetic variation, may advance the impact of clinical genetics studies. Here the Besenbacher *et al*. carry out deep sequencing and *de novo* assembly of 10 parent–child trios to generate a Danish pan-genome that provides insight into structural variation, *de novo* mutation rates and variant calling.

## Graphical abstract

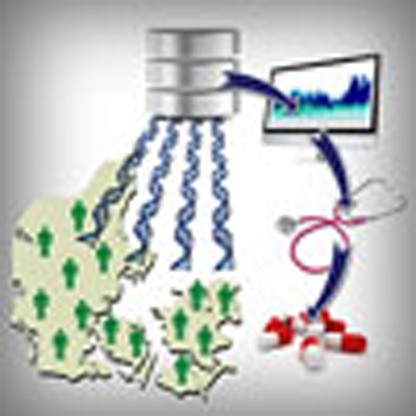

The ability to study human genomes and to discover a complete set of variations between individual genomes has increased tremendously with advances in sequencing throughput and analysis capabilities. Considerable efforts have led to categorization of millions of single nucleotide variants (SNVs), small insertions and deletions (indels) and larger structural variants (SVs) between human individuals and populations^1^,^2^,^3^,^4^,^5^. A population-specific inventory of all detectable variation, a ‘national pan-genome’, has importance for clinical and public health genetics, for example, in facilitating imputation of rare variants in genome-wide association studies and low-pass sequencing studies^6^,^7^ and in addressing missing heritability due to an incomplete or inadequate human reference genome. However, the ability to phase and impute complex variants and haplotype regions relies on accurate identification of these in the reference data. There is a clear trade-off between using available resources for sequencing a few individuals at high quality and sequencing depth or many individuals at lower coverage. Most studies to date have been based mainly on low (4–5 × )^1^ or intermediate (10–30 × )^7^,^8^,^9^ sequencing depth with a few notable exceptions^6^,^10^. Identification of indels and SVs from short reads are challenging and most methods are based on alignment of reads to reference genomes and identifying SVs from irregularities in paired end mapping or local assembly^11^,^12^,^13^,^14^. However, read mapping to regions containing large and complex variants remain troublesome and especially insertions are difficult because a large fraction of the read is novel sequence that is not represented in the reference genome. Higher depth provides more accurate genotyping of variants and long-range libraries allow better characterization of indels and SVs, and a good basis for complementing approaches with genome-wide array scans cataloguing population-specific variation.

High depth and trio information are also needed for estimating *de novo* mutation events. Characterizing *de novo* mutations are fundamental for investigating the causes of genetic diseases^15^ and determining their rate is important for timing events in human evolution^16^. Recent studies have used whole-genome sequencing of trios to directly estimate the SNV *de novo* mutation rate. Given the rarity of the *de novo* events, calculating such an estimate is not a trivial task and there are still some uncertainties about the actual rate and the strength of the paternal age effect^17^. Because *de novo* indels are even rarer than *de novo* point mutations and moreover harder to detect, most studies of *de novo* events have not included these. Currently the only direct measure of indel *de novo* mutation rate comes from a study of a single trio^18^.

Here we present a variant catalogue established through sequencing 10 trios to high depth (50 × ) using libraries with insert sizes from 180 to 800 bp. High quality variants were called and false discovery rate (FDR) was determined to be low in validation experiments. We report 8.37 million bi-allelic SNVs and 1.24 million bi-allelic short indels from alignment-based methods of which 6.4% and 22.8% were novel, respectively. We use the trio structure of the data to identify *de novo* mutations and develop a probabilistic method to determine *de novo* mutation rates for SNVs and indels. For indels we provide the, to date, most accurate *de novo* mutation rate of 1.5e−9 per nucleotide per generation. For SNVs, we estimate a mutation rate of 1.27e−8 per nucleotide per generation, which is slightly higher compared with previous studies. In addition, as *de novo* assembly previously has shown promise for detection of SVs in single human genomes^19^,^20^, we expand this approach to be used at population level and identify 53.2k and 78.5k novel deletions and insertions (>10 bp), respectively, with a low FDR. Medium-sized (20–300 bp) insertions display a high rate of novelty (49.0k of 53.1k are novel; 92.2%) and a low overlap with alignment-based methods (<10%). This is likely due to ascertainment bias when using traditional alignment-based methods for detection of insertions and underlines the importance of *de novo* assembly-based techniques for discovering variation.

## Results

### Discovering novel SNVs and short indel variation

**Figure 1:**
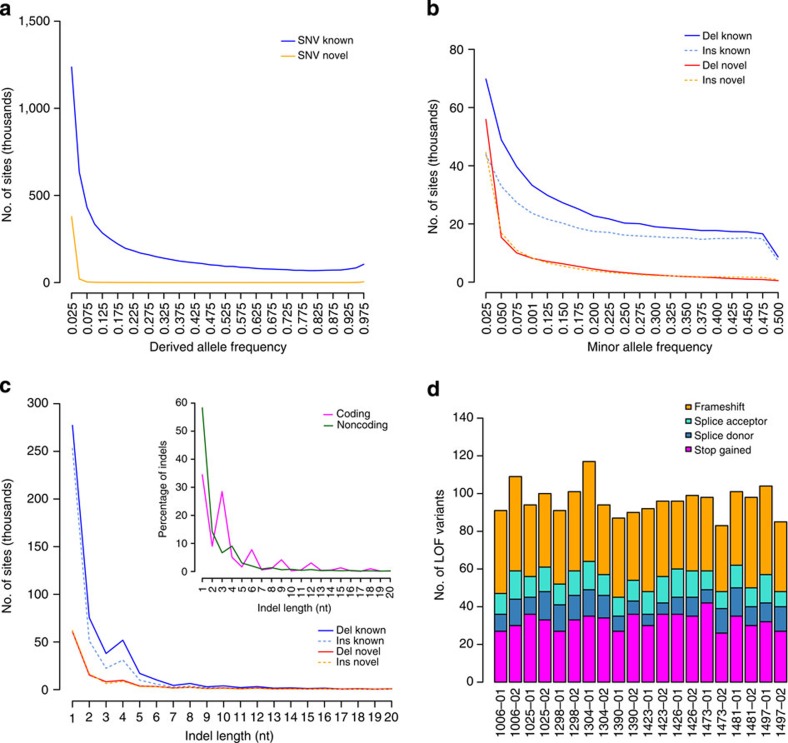
Allele frequencies and loss of function (LOF) mutations. (**a**) Derived allele frequencies of bi-allelic known (*n*=6.69M, blue) and novel (*n*=415k, orange) SNVs with genotype information in all 20 parents. Fixed variants are excluded. (**b**) Folded minor allele frequencies of known deletions (*n*=510k, solid blue), known insertions (*n*=383k, dashed turquoise), novel deletions (*n*=136k, solid red) and novel insertions (*n*=126k, dashed orange) with regard to the reference genome. Only bi-allelic and non-fixed sites with genotype information in the 20 parents are included (for derived allele frequencies, see Supplementary Fig. 1). (**c**) Size distribution of bi-allelic and non-fixed indels (*n*=1.19M) and indels in coding regions only (*n*=1392, insert), legend as in **b**, for insert coding indels (purple) and non-coding (green). (**d**) Estimated number of LOF variants for each parent (*n*=20), in total 10.6% of the mutations was in olfactory genes and 4.1% in zinc finger proteins. Stop gains (magenta), splice donor (blue), splice acceptor (turquoise) and indel frameshift (orange).

Ten father–mother–offspring Danish trios from the Copenhagen Family Bank^21^,^22^ were sequenced to an average of 52 × , except for four individuals sequenced to 19 × (Supplementary Data 1). SNVs and short indels (<50 bp) were called using the Genome Analysis Toolkit (GATK)^23^. Our analyses lead to the detection of 8.37 million bi-allelic SNVs and 1.24 million short bi-allelic indels. The concordance between the sequencing SNV calls and chip genotyping data is 99.8%, which is higher than the 98.9% achieved by intermediate range depth sequencing in a recent study by Boomsma *et al*.^8^ (Supplementary Table 1). We observed 536k SNVs (6.4%) and 283k indels (22.8%) not previously reported and as expected novel variants were generally rare in the population (Fig. 1a,b, Supplementary Tables 2–3, Supplementary Fig. 1). The size distribution of indels shows enrichment of 2 and 4 bp indels outside exons, and that 44% of indels in exons are in-frame (Fig. 1c). Due to the high number of novel indels and that indels in repeat regions are hard to accurately call, we classified the indels based on their primary sequence context into homopolymer runs (HRs) and tandem repeats (TRs)^4^. In total 40.9% of the indels were associated with a canonical HR or TR and an additional 19.3% were associated with non-canonical HR or TR sites. The FDR determined from Sanger sequencing was very low for SNVs (2%), however, indels had a higher overall FDR (15%). The high FDR was due to indels in repeat regions (27% FDR) compared with non-repeat regions (0% FDR) (Table 1 and Supplementary Data 2). We estimated the number of loss of function (LOF) mutations in consensus coding sequences^24^,^25^,^26^ to be in the range 83–117 per individual (average 111.8; Fig. 1d) and individuals were homozygous for 18–33 of these, which is in line with previous findings^7^,^27^. LOF mutations were generally rare with 46% being private to the individuals, suggesting strong purifying selection. Likewise, in-frame indels show evidence of purifying selection (Supplementary Fig. 2). When subjected to experimental validation (Table 1), we determined a FDR of 0 for LOF SNVs (0/25) and 0.13 for LOF indels (2/15). Both of the invalidated indels were located in HRs.

### Calling *de novo* mutations

**Figure 2:**
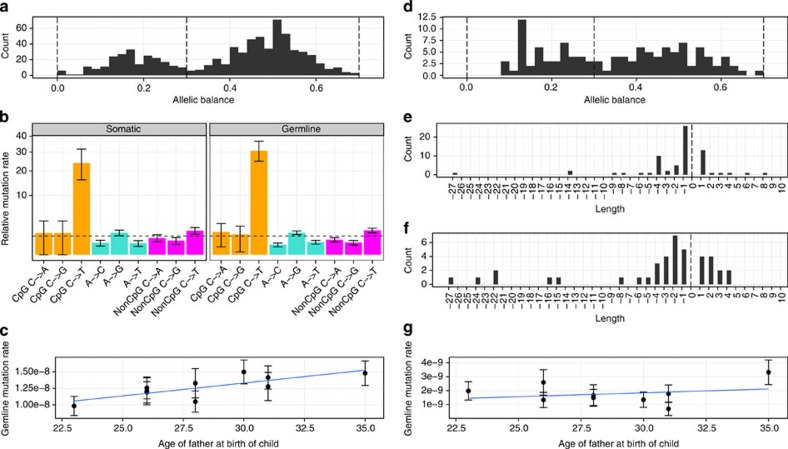
***De novo*** **events in the trios.** (**a**) Allele balance of detected *de novo* SNVs (*n*=730). Variants with low allele balance (<0.3) are considered to be somatic mutations while variants with high allele balance (>0.3) are considered to be germline mutations. (**b**) Mutational context of somatic (*n*=222) and germline (*n*=508) *de novo* SNVs, assuming that there are no strand differences (that is, G->T mutations are considered equal to C->A mutations). Both somatic and germline mutations follow the same pattern of increased frequency of transitions versus transversions and an extremely high transition rate in CpG sites. Orange: CpG mutations, turquoise: mutations at A or T site and magenta: non-CpG mutations at C or G site. Error bars represent s.e.m. (**c**) Germline SNVs increase significantly with paternal age. The blue line is a linear fit to the age of the father at the child’s birth and germline SNV mutation rate, and the error bars represent s.e.m. (**d**) Allele balance of detected *de novo* indels (*n*=121). (**e**,**f**) The indel length distribution indicates that short deletions are more common than short insertions in both germline (*n*=70) (**e**) and somatic tissue (*n*=51) (**f**). (**g**) Germline indel rate show no compelling correlation with paternal age, the blue line is a linear fit to the age of the father at the child’s birth and germline indel mutation rate, and the error bars represent s.e.m.

We identified *de novo* variants by identifying new variants in the offspring that are not present in either of the parents. After applying conservative filters on the quality of the variants and on the genotype quality of the three members of the trio (see methods), we had 730 candidate *de novo* point mutations. Looking at the fraction of the reads in the proband that carry the alternative allele (the allele balance), reveals a bimodal distribution (Fig. 2a) that apart from the expected mode at 50% also has a mode ~18%. Since 98% of SNVs with similar quality show an allele balance between 30 and 70% (Supplementary Fig. 3), we only consider the 508 candidate mutations that fall in this range as being genuine *de novo* germline mutations (applying the same 30% allele balance cutoff as in Kong *et al*.^9^ and Neale *et al*.^28^). We believe that the remaining 222 variants are either somatic variants with low allele balance (only present in a fraction of the sequenced blood cells) or sequencing artifacts. The true number of somatic variants in these individuals is expected to be much higher since recent somatic mutations are unlikely to be detected being present only in a few cells and therefore too infrequent to pass the conservative genotype quality filters. We have called short indel (<40 bp) variants using the same approach and observe 70 germline variants and 54 putative somatic variants. Sanger sequencing validation shows a low FDR for SNVs (4.1%, 1/24), whereas *de novo* indel validation was made difficult by *de novo* indels mostly occurring in highly repetitive DNA (estimated FDR 10.5%, 2/19, Table 1 and Supplementary Data 3).

### Estimation of mutation rates for SNVs and indels

We employed a novel probabilistic approach to estimate the effective number of genomic positions where we would be able to call *de novo* mutations (see methods). We find an average germline mutation rate of 1.27e−8 (95% CI of 1.16e−8 to 1.38e−8) per generation corresponding to ~73 expected *de novo* SNVs in each newborn. The inferred mutation rate in offspring is significantly positively correlated with the age of the father (Fig. 2c). The estimated effect of the father’s age is 3.88e−10 extra mutations per nucleotide per year corresponding to approximately two extra autosomal mutations per year, which is the same effect as was measured in a large Icelandic study^9^. The estimate of the mutation rate per generation depends a lot on the average age of the fathers, which in our study is 28.4 years. Our rate estimate is higher (but within the 95% significance threshold) than reported in the largest similar study of mutation rate (1.2e−8, average age of fathers: 29.7)^9^ and significantly larger than another study with corresponding sample size (1e−8, average age of fathers: 33.6)^29^.

As expected, we find no correlation between the age of the parents (at the offspring’s birth) and the number of somatic *de novo* mutations. Likewise we did not find any correlation between the offspring age and the number of putative somatic variants (Supplementary Fig. 4). We find the mutational pattern to be very similar for germline mutations and putative somatic mutations, with a transition/transversion ratio ~2 for non-CpG sites and an extremely high transition rate for CpG sites (Fig. 2b). Of the 508 germline *de novo* mutations, 18 (3.5%) are already present in database of single nucleotide polymorphisms (dbSNP). Of these 18 mutations, 50% are transitions in CpG sites compared with only 19% of the 490 mutations not in dbSNP, supporting that the overlap is due to recurrent mutations. We estimate the mutation rate of short germline indels to be 1.5e−9 (95% CI of 1.2e−9 to 1.9e−9) per nucleotide per generation, which corresponds to ~9 autosomal *de novo* indels in each newborn. This is consistent with the results of a study that analyzed whole-genome data from a single trio and reached a rate estimate of 1.0e−9 (95% CI of 2.35e−10 to 2.75e−9)^18^ but somewhat higher than an estimate based on the sequencing of Mendelian disease genes (0.78e−9)^30^. We observe approximately eight times more point mutations than indels, which is the same ratio as has been estimated based on whole-genome alignment of the human and mouse genomes^31^. The correlation between paternal age and the rate of germline indels (Fig. 2g) is not significant, perhaps not surprising given the low number of events we observe in each family. The length distribution of the *de novo* indels shows a tendency for a higher deletion rate both for germline and somatic indels (Fig. 2e,f).

### *De novo* assembly of 10 human trios

Preliminary efforts have revealed how *de novo* assembly strategies can be employed for detecting complex human variation^19^,^20^, and here we extend it to be used in a population scenario. Using SoapDenovo2 (ref. 32) we *de novo* assembled the individual genomes to an average N50 of 28 kbp and 12 kbp for scaffolds and scaftigs, respectively, (stretches of non-N bases in the scaffolds; Supplementary Fig. 5). We then aligned the assemblies to the reference genome and after excluding the ambiguous alignments (misalignment probability *P*≥0.01) the individual assemblies covered ~95% of the reference genome (Supplementary Fig. 6, Supplementary Data 4). We observed lower coverage of the assemblies over interspersed repeats, TRs and segmental duplications^33^ (Supplementary Fig. 7, Supplementary Data 4). We identified 10 Mbp of sequences (>100 bp) per individual that could not be aligned to the human reference genome (total across 10 trios 20 Mbp). Most of these sequences (95%) can be mapped to the decoy sequence that contains alternative human assembly sequences (patches to build 37, sequences from HuRef^34^ and NA12878 alternate assemblies), human fosmid clones and Epstein–Barr virus sequence (Supplementary Fig. 8, Supplementary Data 5). The remaining 300 kbp can be mapped to other human genomes (Supplementary Fig. 8). Of the unmapped sequences, 1.2 Mbp can be localized in the reference genome using the chimp genome and flanking sequences in the *de novo* assemblies, most of which are deletions in terms of ancestral state (Supplementary Fig. 9).

### Population scale calling of SVs

**Figure 3:**
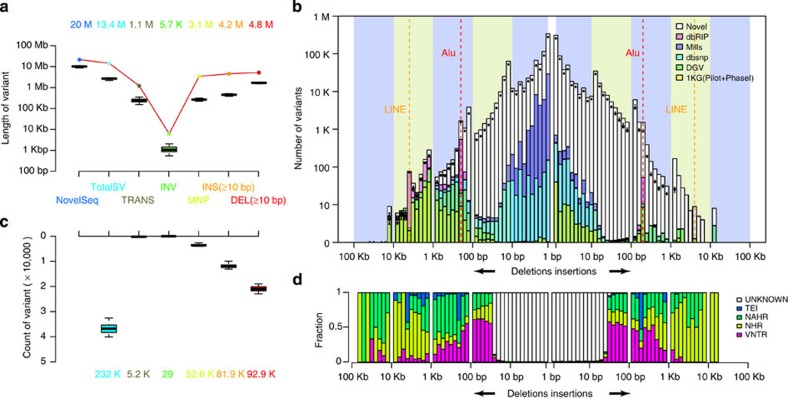
Structural variants and novel sequences identified in the *de novo* assemblies of 10 trios. (**a**) Length of the variants present in the individual assemblies (*n*=30), the total length is given by the coloured numbers. The lower and upper hinges of the boxes correspond to the 25th and 75th percentiles and the whiskers represent the 1.5 × inter-quartile range (IQR) extending from the hinges. See Supplementary Fig. 10 for definitions of different types of structural variants. (**b**) Same as **a** but count of variants instead, individual counts are shown as box plots and total count by coloured numbers. (**c**) Length distribution and novelty of the variants (*n*=232k, 50% reciprocal overlap). The box plots indicate the number of variants per individual (*n*=30) at a certain length range; see box plot definition in **a**. Red dashed line: Alu peak at 300–400 bp. Orange dashed line: LINE peak at 6–7 kbp. (**d**) Variant mechanism. The *y*-axis indicates the proportion of variants annotated with different mechanisms corresponding to the length range in **c**. NAHR, non-allelic homologous recombination (green); NHR, non-homologous rearrangement (yellow); TEI, transposable element insertion (blue); unknown (white); VNTR: variable number of tandem repeats (magenta).

We developed the Soap Assembly Variation discovery pipeline (SoapAsmVar, see methods and Supplementary Fig. 10) and employed it to detect SVs in the individual *de novo* assemblies. The identification of the SVs was performed at per individual level, combined to population level and thoroughly filtered (see methods). We identified a variety of SVs (232k) including deletions (81.9k, ≥10 bp), insertions (92.9k, ≥10 bp), multiple nucleotide polymorphisms (52.6k), inversions (29) and translocations (5.2k; Fig. 3a,b). The number of these events is only a fraction of the SNVs (232k versus 8.3 million), but the number of base pairs affected by the non-redundant set is 13.4 Mb, almost two times the number of SNVs, consistent with previous study of the HuRef *de novo* assembly^34^. The number and length distribution of the insertions and deletions are highly symmetric and is consistent with previous studies of a few individual *de novo* assemblies^20^,^34^ (Fig. 3c). A substantial part of the SVs are previously unknown using a 50% reciprocal overlap criteria (insertions: 78.5k, 84.5% and deletions: 53.2k, 64.9%) and these variants were enriched in 10–200 bp for deletions and almost all length ranges of the insertions. This enrichment was especially large for insertions of length 20–300 bp of which 92.2% were novel (49.0k of 53.1k in total) and to a lesser degree for deletions in this range (23.0k of 30.9k in total, 74.4%). The size spectrum corresponds to distinct formation mechanisms and is symmetric between deletions and insertions^3^ (Fig. 3d). Variants within 50–200 bp tend to be associated with variable number of TRs (VNTR), while variants of 300 and 6 kbp derive from transposable element insertions. Most of the larger variants (>1 kbp) are related to non-allelic homologous recombination (NAHR) or non-homologous recombination (NHR). As VNTR indels can be hard to accurately call^3^ we benchmarked the VNTR calls by investigating the Mendelian errors of this variant category. In total 25.4% of the raw 25,363 VNTRs contained at least one Mendelian error and 8.1% of them contained at least two Mendelian errors prior to filtration using other metrics. However this was not different compared with the Mendelian error rate among the other raw SoapAsmVar calls and displayed less Mendelian conflicts compared with raw indel calls from GATK (Supplementary Table 4).

We compared the SoapAsmVar variants to the variants called by GATK and found that for very short indels (1–4 bp) 65% of the GATK calls were also called by SoapAsmVar and that 50% of SoapAsmVar indels in this range were called by GATK (Supplementary Fig. 11). However, when increasing the length of the variants to 50 bp the fraction of SoapAsmVar calls which were also called by GATK declined to 38% for deletions and 25% for insertions. The genotype concordance at the overlapping sites was high ranging from 57.7 to 76.7% (Supplementary Fig. 11). The difference in concordance between the call sets reflects the difficulties in defining complex variants in the human genome and *de novo* assembly may represent a promising alternative strategy for variant discovery^35^.

Because of the high novelty of the SoapAsmVar callset and that SoapAsmVar called 19 times more insertions (≥50 bp) and 4.5 times more deletions (≥50 bp) compared with GATK we experimentally validated a subset of the novel variants (Table 2, Supplementary Data 2). We observed an overall FDR of 7.3% (5/68) of which the FDR was higher for deletions (17%) compared with insertions (5%). When investigating the mechanism of formation especially indels associated with NAHR had a high FDR (22%), however, the assay was only successful for nine sites. In general the success rate of performing the validation assays was relatively low and only 68 of the 272 randomly selected variants could be assayed (Supplementary Data 2). This was particular true for the VNTR validation assays where we were unable to perform any of them due to their repetitive structure (25 were randomly selected)^3^. This emphasizes the difficulties caused by the sequence context in correctly calling and assaying SVs and the FDR for difficult sites may therefore be higher than what we report.

## Discussion

Constructing a national pan-genome requires a variant catalogue as complete and as little biased against complex variants as possible. We have shown that a strategy based on high depth sequencing, local assembly and *de novo* assembly can identify novel SNVs, indels and SVs with high accuracy as estimated from Sanger sequence validation experiments. Identification of indels and SVs are critical to assess the full variability of individual genomes, however, current methods are mainly based on alignment of reads to a reference genome, which is not a very powerful way of detecting large and complex variants.

**Figure 4:**
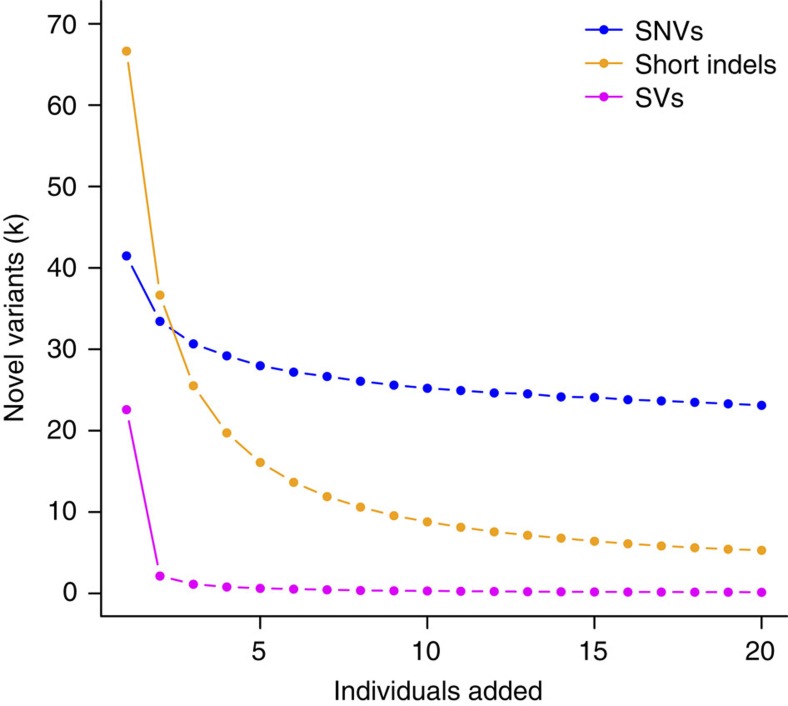
Number of novel variants per sample. Number of novel variants identified from adding additional unrelated individuals (*n*=20). The visualized data is the average of 1,000 random samples of the individual order. Blue, SNVs; magenta, SVs >50 bp; orange, short indels.

Utilizing a population based *de novo* assembly approach such as SoapAsmVar greatly facilitates the identification of such variants because the *de novo*-assembled sequences are much longer (scaffold N50: 28 kb) and therefore easier to anchor using sequence that flank the variants. This is exemplified by the medium-sized insertions (20–300 bp) of which 92.2% of the variants were novel. In contrast, only 6.4% of the SNVs that we identify have not been reported previously, because previous efforts have primarily targeted SNVs and to a lesser extent short indels. Our approach is powerful for identifying a much wider set of novel variants for short indels and particularly longer indels and SVs. We have estimated a low FDR of the identified variants but estimation of the sensitivity for the SVs will require longer insert libraries or longer read lengths. As expected, adding more individuals to the pan-genome will increase the number of variants (Fig. 4). For indels, a high proportion of novel variants are found in the first individual, reflecting that even very common SVs have not been previously identified. In contrast, novel SNVs are mainly rare variants or variants that have an exclusively high frequency in the Danish population.

Obtaining accurate *de novo* mutation rate estimates is a key factor for understanding human evolution^16^. The estimation of mutation rates is more challenging than the identification of the *de novo* mutations themselves as it requires an accurate estimation of the fraction of the genome where the *de novo* events could be observed (the denominator of the mutation rate estimator). Using a novel probabilistic approach, which estimates the effective number of genomic positions where we can call *de novo* mutations, we estimate the rate for *de novo* SNV germline mutations and make a more precise estimate of indel mutation rates from direct sequencing. The differences from the previous rate estimates^9^,^18^,^28^ are mainly caused by differences in the way the denominators are calculated rather than differences in the number of mutations per individuals that are found in the studies. We estimate a rate that is higher than most previous estimates, making it slightly closer to mutation rates estimated from phylogenetic estimates. We also show that it is possible to find short *de novo* indels, which may play an important role in disease.

Identification of all variants and their frequencies can facilitate an increased understanding of population-specific disease susceptibility and will be important for advancing clinical and public health genetics^7^,^36^. In addition, future efforts should be aimed at building a true national pan-genome sequence that can replace or augment the current reference genome for national sequencing projects. This will require either longer reads or the use of long-range mate pair libraries to produce long scaffolds, which can improve gaps in the current reference genome.

Methodological developments in the analysis and representation of sequence data will offer advantages as well as addressing challenges such as storing of variant population frequencies and alternative haplotypes within a reference possibly through a population sequence graph. Population-wide *de novo* assembly will certainly be needed to facilitate discovery of complex variants, and the vast range of SVs identified in this project indicates their importance for our understanding of the structure of the human genome.

## Methods

### Cohort selection

The 10 trios (mother–father–child) for the pilot study of the Genome Denmark project were selected from the Copenhagen Family Bank^21^,^22^. The Copenhagen Family Bank dates back to the 1970s and constitutes a reference databank for linkage analysis as it archives sampled blood from families with numerous children together with comprehensive information about phenotypic traits. We selected the individuals as part of a pilot effort for a larger study using the criteria that (1) the trio individuals are still alive, (2) individuals reside in the Copenhagen area, (3) have provided informed consent for further participation and (4) there was enough blood available for DNA extraction and library preparation. After sequencing we discovered that individual 1006-01 was of half Greenlandic ancestry. We decided to include the trio in the analysis due to the Danish–Greenlandic history and number of individuals resident in Denmark that are of Greenlandic decent. All participants provided informed consent and the study protocol was reviewed and approved by The Danish National Committee on Health Research Ethics file no: 1210920, submission numbers 36615 and 38259.

### Library construction and sequencing

Library construction and sequencing was performed by Illumina HiSeq 2000 following the manufacturer’s instruction. Base calling was performed using CASAVA 1.7. For each individual, three small insert size libraries were constructed and sequenced—180 bp (30 × ), 500 bp (10 × ) and 800 bp (10 × ).

### Chip genotyping

Among the 10 trios, three subjects did not have enough DNA left for genotyping. For the remaining 27 subjects, HumanCoreExome BeadChips were used on a HiScan system (Illumina, San Diego, CA, USA), and the genotypes were called using GenomeStudio software (version 2011.1; Illumina). All subjects had a high call rate (>98%), and the familial relationship and the sex of the subjects were confirmed. SNPs with a low call rate (<98%) or deviation from Hardy–Weinberg equilibrium (*P*<0.0001) were excluded.

### Read trimming and correction

After initial quality control assessment with FastQC version 0.10.1 (ref. 37), AdapterRemoval^38^ was used to trim the tails of the reads if the Phred quality dropped <2. AdapterRemoval was also used to collapse 180 bp insert size libraries into longer single end reads.

### Alignment-based assembly

All reads from the compendium of libraries were mapped to the human reference genome build 37 supplemented with unlocalized contigs and the decoy sequence using BWA-MEM; version 0.7.5a (ref. 39). SAMtools version 0.1.19 (ref. 40) and Picard version 1.96 (http://picard.sourceforge.net) were used to process the alignment files and to mark duplicate reads. GATK version 2.7-2 (ref. 23) was used to refine the alignments by performing local indel realignment and subsequent base quality recalibration using Mills_and_1000G_gold_standard indels and NCBI’s dbSNP (build 138) as known variant sites. Duplicate marking and base recalibration was performed at lane level BAMs. Local indel realignment was performed both for each individual lane as well as after merging BAMs by sample.

### Genotyping

The Base Quality Score Recalibrated BAM files of the 30 individuals were used as input for multi-sample genotyping using the HaplotypeCaller of the Genome Analysis Toolkit version 2.7.2 (ref. 23). The raw variants were recalibrated using VariantQualityScoreRecalibration (VQSR) including HapMap, Omnichip, 1000G phase 1 high confidence variants and dbSNP138 using arguments given by best practices by the Broad Institute^41^. Furthermore the following annotations were used: QD, MQRankSum, ReadPosRankSum, FS and DP as well as ‘--numBadVariants 5000’. The indels were recalibrated using Mills and 1000G database^5^ using similar parameters as above except for ‘--maxGaussians 4 --numBadVariants 1000. The SNVs were filtered at a truth-sensitivity tranche of 99.5, whereas the indels were filtered at 95.0. Genotype concordance of the called genotypes to HumanCoreExome calls was performed using PLINK^42^ removing sites with AT and CG alleles.

### Site frequency spectrum and variation effect

Derived alleles were determined from the Ensembl compara v71 EPO alignments using only high confidence calls and only bi-allelic sites with genotype calls for all individuals were used for the site frequency spectrums. The SNVs and indels were annotated for their effect on the proteins using variant effect predictor tool from Ensembl version 73 (ref. 43). To identify LOF mutations we used only proteins consensus coding sequences^24^,^25^,^26^ and disregarded mutations if (a) the mutation was fixed in the samples, (b) the variant allele was the same as the ancestral state based on human-primate alignments, (c) if the variant was in a non-canonical splice site or (d) the variant occurred in the first or last 5% of the gene. Filters b–d are similar to what was used in refs 7, 27. For indels, ancestral alleles were identified by extracting ±10 nt around the indel and manual inspection of presence or absence of the indel in the ancestral allele. Novel SNVs were determined from dbSNP138, and novel indels were determined as not in dbSNP138, and not found in Mills and 1000G database^5^ or Database of Genomic Variants^44^ with 50% reciprocal overlap.

### Indel repeat classification

The sequence 100 bp upstream and downstream of the outer indel coordinates were used as input to Tandem Repeat Finder (TRF)^45^ and repeat annotations spanning the indels were extracted. Indels were classified as a canonical HR if the variant was within a run of six or more identical bases, as a TR if the variant was within a segment of at least two repeated sequences >1 bp. A TR indel was annotated as canonical if the repeated segment was recurring at least UnitLength × 2+5 times, for example, a repeat segment of 2 bp must be repeated at least 9 times, a repeat segment of 3 bp must be repeated at least 11 times and so on^4^. Variants where the HR consisted of not only the HR base where classified as non-canonical HR and variants where the TR did not fulfil the minimum repeated number were classified as non-canonical TR. The VCF file contains the annotations in the info field as HR, TR, HR_NC and TR_NC.

### Identification of *de novo* SNV

We developed a new method for detecting *de novo* mutations, where we incorporate sequencing depth as a variable and not as a strict filter. To limit the number of false positives we only consider a Mendelian violation as a possible *de novo* mutation if both parents in the family in question are homozygotes for the reference allele and if the variant is not called in any of the other families.

We apply the following filters when we look for *de novo* mutations:

1. A site filter that looks at the reads from all 30 individuals to filter away bad sites that are not true variants. The site filter uses the following tests:
2. Individual filters that look at the reads and genotype call of a single individual to discard a possible *de novo* call if we are not sure that all of the individuals in the family in question are called correctly. We use two different kinds of Individual filters:

### Estimating callable sites for *de novo* mutations

To estimate the rate of *de novo* mutations in a trio, we base the denominator of the rate estimate on the probability at each site that we can call *de novo* mutation rather than simply counting a site as either callable or non-callable. The probability of calling site *x* as a *de novo* mutation given that it is a true *de novo* mutation in the family *f* we name the callability and denote it by 
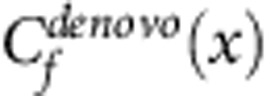
. The callability can be estimated independently for each family based on the depth of the family members at the site, and the expected number of callable sites in a given family is then the sum of the callability of all sites in that family.

Since the site filter is based on statistical tests that follow a known distribution, we can estimate how many good sites we expect to be filtered away by this filter. For this purpose we look at the null distribution of the tests and assume that the two tests are independent. We denote by *α*_site_ the fraction of good sites that we expect to be filtered away.

The mutation rate of a family f can then be estimated as:


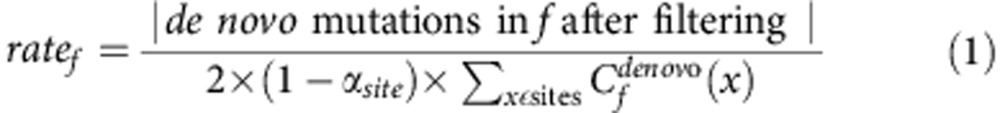


Now let *Z* be a genotype (Hetero, HomRef or HomAlt) and consider for an individual *i* the probability of calling it as *Z* at position *x* (and not filtering it away) given that the individual truly is *Z* at *x*. We denote this conditional probability by 
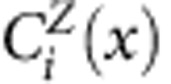
 and it signifies the ability to give a true call of *Z* at *x*. Clearly this will be a function of sequencing quality at *x* (not least the depth). If we assume that the ability to truly call each member of a family is independent, then the callability of a site in a given family can be calculated as the probability of calling each individual correctly after filtering:


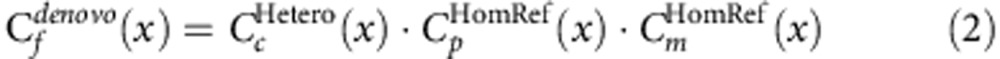


where *c, p* and *m* indicate the child, father and mother of the family *f*.

Assuming that 
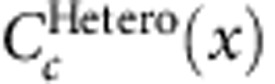
 is independent of the parental genotypes as long as they are conducive to a heterozygous offspring, we can estimate it by considering only variants where one parent is homozygous reference with high confidence and the other parent is homozygous for the alternative allele. At such sites the child should always be a heterozygote (barring *de novo* events). Using these sites only we can estimate:


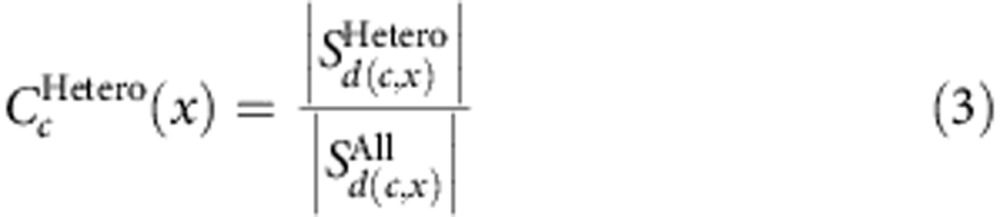


where *d*(c*,x*) is the depth at *x* for the child c and 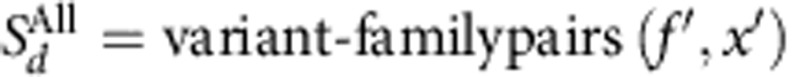 where the child c′ has depth *d*(c,*x*)=*d* at variant *x*′ and one of the parents is HomRef from the variant and the other parent is HomAlt after applying the sites filter and a conservative filter on the genotype quality of the parents,


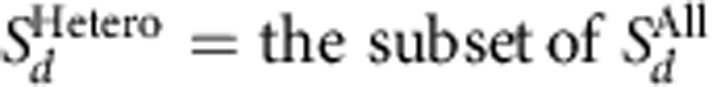
 where the child is called as heterozygous and passes the heterozygote filter.

Similarly we can calculate:


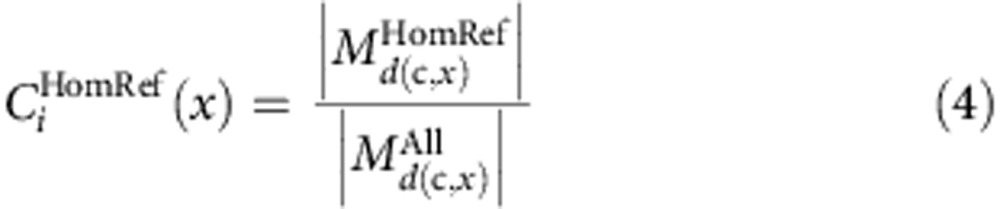


Where *i* is either *m* or *p* and 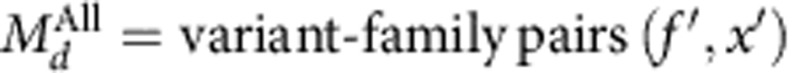 where all the children c′ have depth *d*(c,*x*)=*d*, and both parents in each family in question are HomRef for the variant, the variant is present in at least one of the other families after applying the sites filter and a conservative filter on the genotype quality of the parents, and in the case of SNVs the variant must be in dbSNP,


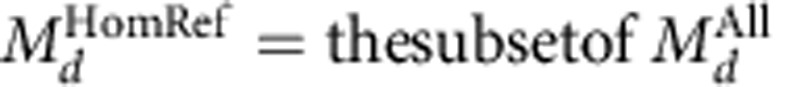
 where the children are called as homozygous for the reference allele and pass the homozygote filter.

Supplementary Fig. 12 shows 
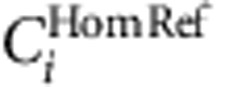
 and 
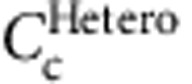
 (for the filter cutoffs described in the next section).

### Minimizing false-positive *de novo* mutation calls

While the estimation of callability, as described above, reduces the effect of false negatives on the estimated mutation rate, it is still necessary to set the cutoffs in the filters so high that only very few or no false positives get into the set of estimated *de novo* mutations. We can fit the filter criteria by looking at the effect of different criteria on the rate estimate and the effect on how large a fraction of the called *de novo* variants are present in dbSNP (Supplementary Fig. 13).

Based on these considerations we set the filter values at:

- GQ≥50 (for both the homozygote and heterozygote filter)
- DPε[10;120] (for both the homozygote and heterozygote filter)
- AD2=0 (for the homozygote filter)
- Allele Balance ε[0.3; 0.7]

The AlleleBalance filter was set based on the distribution of AlleleBalance in the children after applying the other filters (see Fig. 2a).

### *De novo* assembly of the individual genomes

We used the SoapDenovo2 package^32^ to *de novo* assemble each of the 30 individuals. The workflow included (1) data filtration where reads with >40% low quality base or 10% N are removed. (2) error correction where base and indel errors in a read are corrected. (3) connection of 180 bp paired-read reads into 180 bp long reads to improve the gap filling procedure. (4) PreGraph where a *de Bruijn* graph is constructed using *k*=45. (5) contig building where we remove tips and merge bubbles whenever the difference between the two paths is <3 bp, resolve repeats in the *de Bruijn* graph and output the consensus sequence. (6) mapping of the pair-end reads towards the contigs to construct the linkage graph of the contigs sequences. (7) stepwise scaffolding where the contigs that are unambiguously connected by more than five reads are placed in the same scaffold and (8) gap filling where we perform local assembly to iteratively fill the gaps within the scaffolds using all the relevant reads.

### Assembly-versus-assembly alignment

We applied the last aligner^46^ to align the scaffolds to the human reference genome. Split alignment was performed to allow for the existence of genome rearrangements. The misalignment probability was computed providing the Phred-scale confidence of the correctness of genome-scale alignment and the base-scale alignments. In the final assembly-versus-assembly alignments, every non-overlapping DNA piece of the scaffold was anchored to a unique position in the reference and we only kept alignments with misalignment probabilities <0.01.

### Assembly evaluation

We evaluated the individual assemblies using three metrics: continuity, coverage and accuracy. For continuity, we computed the N10 to N90 of the raw contig, scaffold and scaftigs. We also evaluated the proportions of Encode 18 gene and coding regions that could not be covered entirely by one continuous scaffold. The coverage was calculated as the proportion of the bases in the reference genome that were covered by the individual assembly after excluding alignment ambiguity. The accuracy was empirically evaluated by the number of variants (identified in the SVD module in the SoapAsmVar package as described below) that did not obtain support from local realignment or from the short read alignments.

### Variant discovery and genotyping based on *de novo* assemblies

We developed the SoapAsmVar package to discover and genotype the variants from the individual *de novo* assemblies. There are six modules: (1) structural variation detector (SVD) module where we detect and characterize the SVs into ‘indels’, ‘deletions’, ‘insertions’, ‘multiple nucleotide polymorphisms’, ‘duplications’, ‘inversions’ and ‘translocations’ by enumerating the anomalous alignments between the individual assembly and the reference. (2) align-gap-excise (AGE) module where we apply the align-gap-excise algorithm^47^ to validate the variants and to refine both the types and the breakpoints of the variants identified in the SVD module. (3) SVVerified module where we remove potential false-positive calls at which either no significant anomalous short-read-versus-reference alignments or excessive anomalous short-read-versus-assembly alignments is observed. (4) genotype module where we first integrate all the variants from the population and genotype the variant in each individual. For each variant locus, we fit the normalized read depth of the proper aligned reads around the variant loci into a linear constraint Gaussian model and obtain the genotype likelihoods and Phred-scale genotype quality for all the three genotype states. (5) de-duplicate module where we obtain the best alleles for a polymorphic loci when individual assemblies emit different alleles for this loci. (6) posterior treatment module is population based: We keep the variants for downstream analysis that (1) are recurrently observed in more than one individual assembly; (2) for which 50% of the individuals have genotype quality >30; (3) for which the InbreedingCoefficient is >−0.15; (4) do not violate Hardy–Weinberg equilibrium with significance threshold 0.001; (5) do not violate Mendelian inheritance law.

### Novel sequences analysis

We identified the assembled sequences that were >100 bp and that cannot be aligned to the GRCh37 human genome sequence. We realigned the sequences and obtained the novel sequences that were unambiguously aligned to the decoy sequence in 1KGP project, YH assembly, African assemblies and the *Homo sapiens* sequences in the NT database using either last^46^ or blastn^48^.

### Formation mechanisms of the SVs

We applied the breakSeqv1.3 pipeline^3^ to characterize the SVs that were >50 bp into four categories of mechanisms VNTR (Variable number tandem repeat), NAHR (Non-Allelic Homolog Recombination), TEI (Transposonable Element Insertions) and NHR (Non-Homologous Recombination). The ancestral state of the polymorphic loci was determined by comparisons of the reference allele, as well as the observed alternative allele with the chimpanzee (panTro4), orangutan (ponAbe2) and macaque (rheMac3) sequences in the syntenic regions of the corresponding net alignments. The allele with identity and coverage >90% was determined as the ancestral state.

### Validation of polymorphisms

We randomly selected 50 *de novo* SNVs, 50 *de novo* indels, 49 LOF SNVs, 53 LOF indels, 50 novel SNVs, 50 novel indels and 272 novel SVs from SoapAsmVar (>50 nt) covering different size and predicted formation mechanism spectrums for experimental validation using Sanger sequencing. All variants were selected from the 1298 trio and assayed in the father, mother and child. Successfully amplified PCR amplicons was sequenced using a Sanger AB3730xI DNA Analyzer and chromatograms were analyzed using PolyPhred 6.18 (ref. 49) to genotype SNVs and small indels. SVs were analyzed manually. Hereafter all calls were manually inspected using Chromas 2.11, and we required that the variant calls from the NGS pipeline had the exact same breakpoints to be successfully validated.

## Author contributions

The study was conceived and designed by K.K., S.Br., M.H.S., A.D.B., L.B., O.P., T.I.A.S., J.W. and R.G. with contributions from J.G. and N.L. The analysis was headed by M.H.S., P.V., S.R., A.D.B., S.Br., K.K., A.K. and R.G. with input from J.W., O.P., T.H., T.I.A.S. Samples from Copenhagen Family Bank database were typed for classical markers and donated by H.E. Database work and sample selection were performed by H.E., J.M.G.I., K.B., E.R., K.R., D.W., S.Liu and P.C. Sample management, sequencing and analysis was headed by R.X., J.S. and X.X. H.L. performed sample management and sequencing. Sequence data processing and analysis was performed by S.Be., S.Liu, J.M.G.I., J.B.-J., T.D.A., K.B., S.H., S.Li., R.Y., A.R.-G., F.L., J.R., W.Y., T.M., R.M.F., C.N.S.P., D.F., J.D.S., D.W., P.V. and S.R. J.B.-J. headed the chip genotyping and processed the data. The genotype data was analyzed by J.B.-J., T.D.A., S.Liu, W.Y., K.B. and S.R. Analysis of GATK-SNVs and indels was done by J.M.G.I., K.B., R.Y., A.R.-G., R.G., P.V., S.Be. and S.R. Analysis of *de novo* mutations was performed by S.Be., J.G., F.L., P.V. and M.H.S. The *de novo* assemblies and SoapAsmVar development was done by S.Liu, S.H., J.R. and W.Y. Polymorphism validation was performed and analyzed by D.D., P.V., S.Be., S.Liu, W.Y., O.W., and X.C. The manuscript was written by S.Be., S.Liu, J.M.G.I., M.H.S., R.G., P.V. and S.R. with critical input from J.B.-J., K.B., T.D.A., J.G., F.L. A.K., L.B., T.I.A.S. and the remaining authors.

## Additional information

**How to cite this article:** Besenbacher, S. *et al*. Novel variation and *de novo* mutation rates in population-wide *de novo*-assembled Danish trios. *Nat. Commun.* 6:5969 doi: 10.1038/ncomms6969 (2015).

**Accession codes:** SNV and short indel (≤50 nt) data have been deposited in the European Variation Archive (EVA) under the accession code PRJEB7725. SV (>50 nt) data have been deposited in the Database of Genomic Variants archive (DGVa) under the accession code estd217. Whole-genome sequence data for the 10 Danish trios are available under data access agreement from the Genome Denmark access committee via S.R.

## Supplementary Material

Supplementary Figures and Supplementary Tables Supplementary Figures 1-13 and Supplementary Tables 1-4

Supplementary Data 1 Overview of Illumina sequencing data

Supplementary Data 2 Validation of LOF SNVs, LOF indels, novel SNVs, novel indels and novel SVs in 1298 trio

Supplementary Data 3 Validation of de novo mutations

Supplementary Data 4 The de novo assembly coverage and depth of the chromosomes in the reference (Non-N portion)

Supplementary Data 5 Novel sequence distribution in different human assemblies and primate assemblies

## Figures and Tables

**Table 1:** Sanger validation of SNVs, short indels and *de novo* variants.

**Variant class**	**FDR**	**Sites**
*GATK-SNVs*		
Overall	0.02	46
Novel	0.05	21
Loss of function	0.00	25
		
*GATK-indels*		
Overall	0.15	26
Repeat	0.27	15
Non-repeat	0.00	11
Novel	0.18	11
Loss of function	0.13	15
		
*De novo variants*		
SNVs	0.04	24
Indels	0.11	19

FDR, false discovery rate; GATK, Genome Analysis Toolkit; SNV, single nucleotide variation.

Sanger sequencing experiments were used to assay the FDR for 21 novel, 25 loss of function and 24 *de novo* SNVs. For indels a total of 11 novel, 15 loss of function and 19 *de novo* indels were assayed.

**Table 2:** Sanger validation of novel SVs called by SoapAsmVar.

**Variant class**	**FDR**	**Sites**
*Structural variants*		
Novel SVs	0.07	68
		
*By length (bp)*		
50–100	0.10	39
101–300	0.00	9
301–500	0.10	10
501–1,000	0.00	10
		
*By type*		
DEL	0.17	12
INS	0.05	56
		
*By mechanism*		
Unknown	0.04	25
NAHR	0.22	9
NHR	0.06	33
TEI	0.00	1
VNTR	NA	0

DEL, deletion; FDR, false discovery rate; GATK, Genome Analysis Toolkit; INS, insertion; NA, not applicable; NAHR, non-allelic homologous recombination; NHR, non-homologous recombination; SV, structural variant; TEI, transposable element insertions; VNTR, variable number of tandem repeats.

In total 68 novel variants were subjected to validation and the FDR was stratified by length, type of indel and formation mechanism.
