# Supplementary material for: Novel variation and de novo mutation rates in population-wide de novo assembled Danish trios
Source: Nat Commun. 2015 Jan 19;6:5969. doi: 10.1038/ncomms6969 (PMC4309431; doi:10.1038/ncomms6969)
Supplement: Supplementary Figures and Supplementary Tables — Supplementary Figures 1-13 and Supplementary Tables 1-4 [file ncomms6969-s1.pdf]

Supplementary Figures

Supplementary Figure 1: Derived frequency spectrum of deletions compared to ancestral state

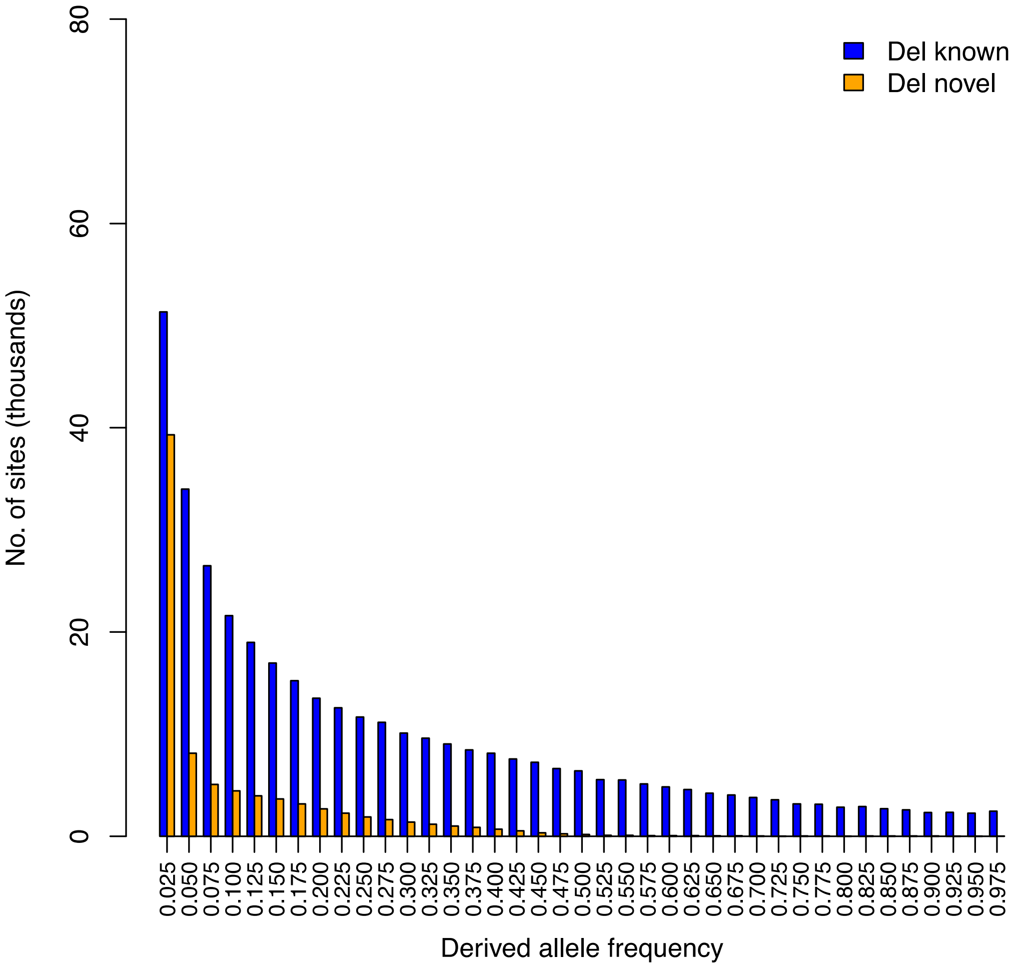

Supplementary Figure 1. Derived allele frequency of known deletions (blue) and novel deletions (orange) in the parents (n=20). Only deletions relative to the ancestral state is shown, e.g. deletions that based on the ancestral alignments are insertions in the reference genome is not shown.

Supplementary Figure 2: Site frequency spectrum of variation by functional class

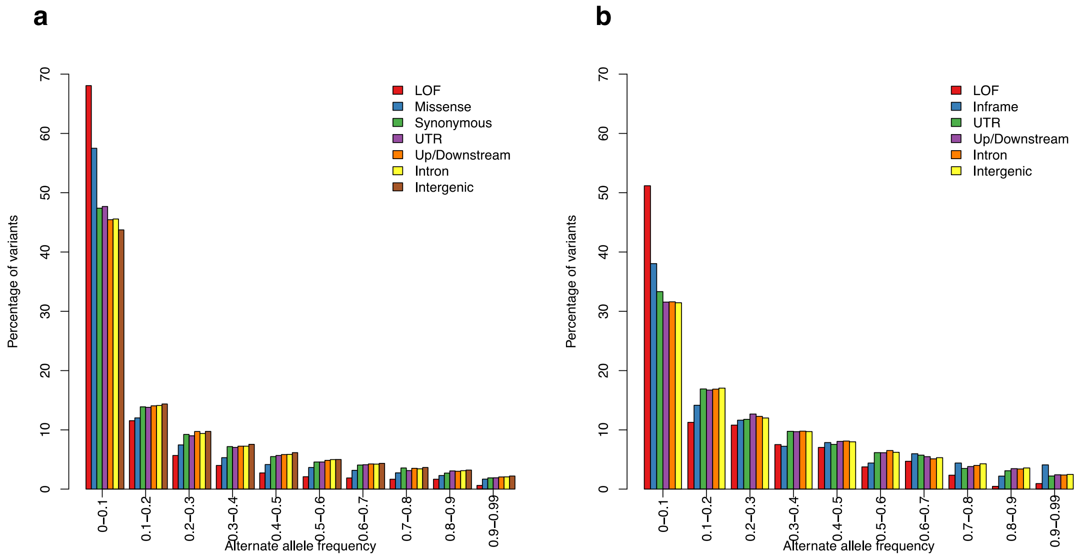

Supplementary Figure 2. Alternate allele frequency spectrum of SNVs (a) and indels (b) identified in the parents (n=20) divided by functional class. For SNVs colors are loss-of-function (red), missense (blue), synonymous (green), UTR (purple), up/downstream (orange), intron (yellow), intergenic (brown). For indels colors are loss-of-function (red), inframe (blue), UTR (green), up/downstream (purple), intron (orange), intergenic (yellow).

Supplementary Figure 3: Allele balance in high confidence heterozygous variants

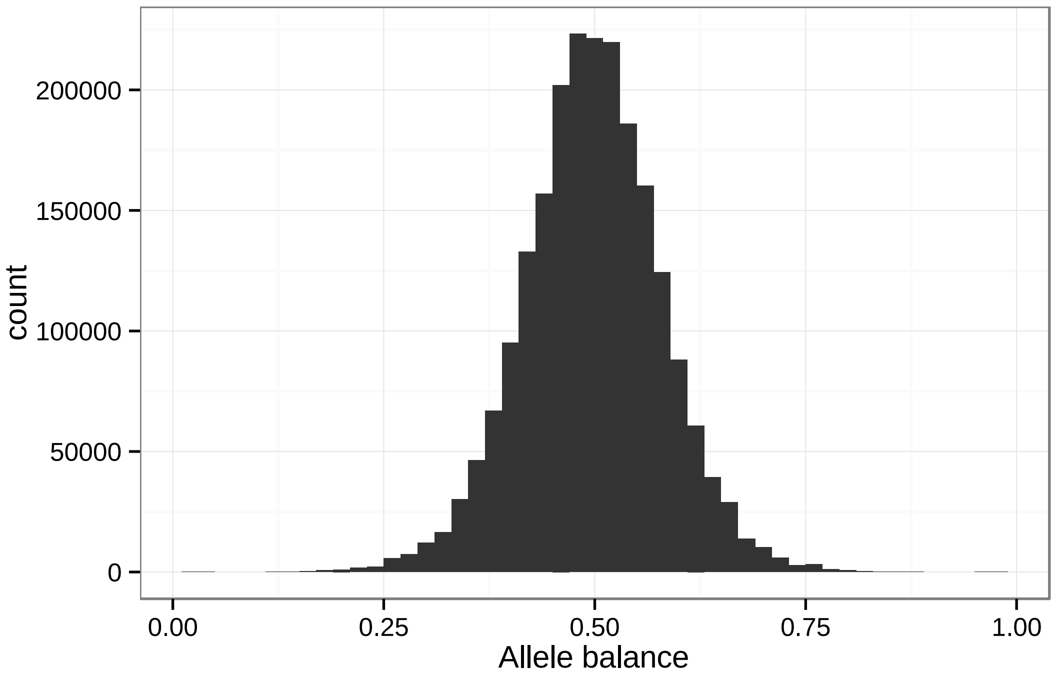

Supplementary Figure 3. The allele balance in children (n=10) with one parent that is homozygous for the reference allele and one parent that is homozygous for the alternative allele. Furthermore all individuals pass the quality filter used when calling *de novo* variants, except the allele balance filter. 98.0% of the variants are in the interval between 30% and 70%.

Supplementary Figure 4: Age effect on the number of somatic de novo SNVs

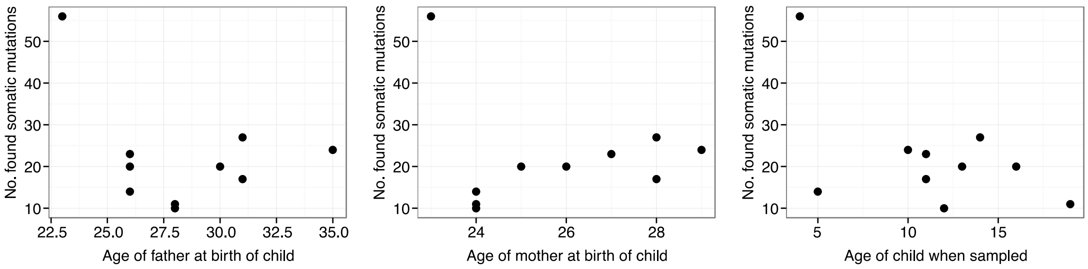

Supplementary Figure 4. The number of somatic SNVs per proband (n=10) plotted versus paternal age, maternal age and the age of the proband when the blood was sampled.

Supplementary Figure 5. Continuity of the de novo assemblies

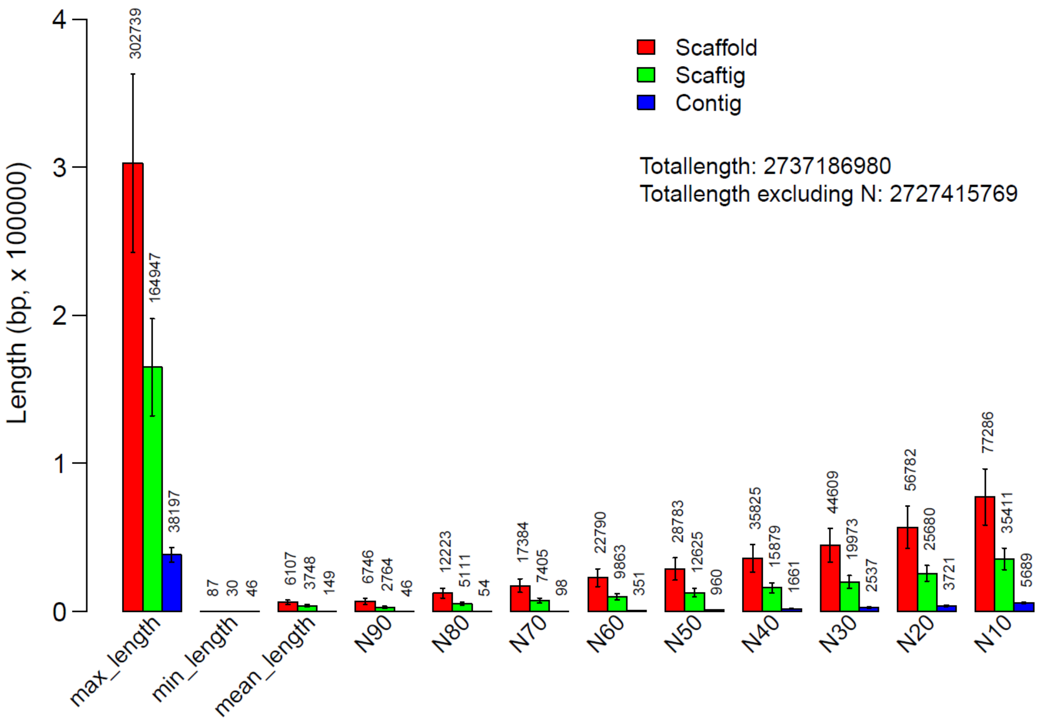

Supplementary Figure 5. Continuity of the *de novo* assemblies (n=30) showing N10-N90 of the raw contigs, scaffolds and scaftigs. Raw contig refers to the unambiguous path in the *de Bruijn* graph before scaffolding; Scaftig: contigs that are cut out from the scaffolds at Ns. N50: after sorting the raw contig/scaffold/scaftig based on the length of raw contig/scaffold/scaftig respectively, N50 refers to the minimum length that the assembled sequences with the length above that comprise 50% of the contig/scaffold/scaftig. The error bars indicataes standard deviation (s.d.).

Supplementary Figure 6. Assembly coverage and depth

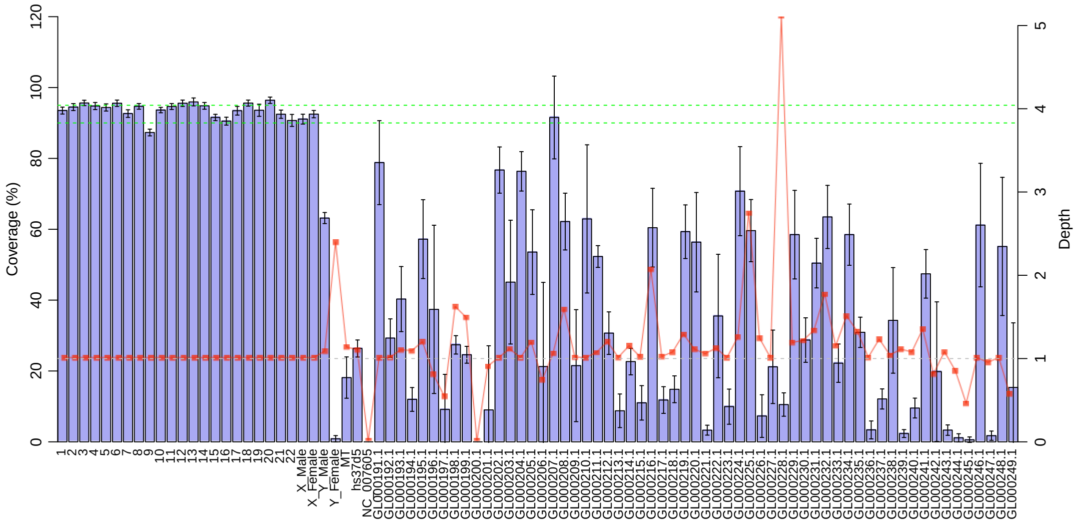

Supplementary Figure 6. Assembly coverage and depth of the chromosomes of the *de novo* assemblies (n=30) when aligned to the 1KGP PhaseII reference. The left y-axis and blue bars indicate the coverage of the chromosomes. The right y-axis and the red lines indicate the depth of the chromosomes. Error bar indicates standard deviations between the 30 individuals. Green dashed lines indicates 90% and 95% coverage, respectively.

Supplementary Figure 7. Assembly coverage of genomic features

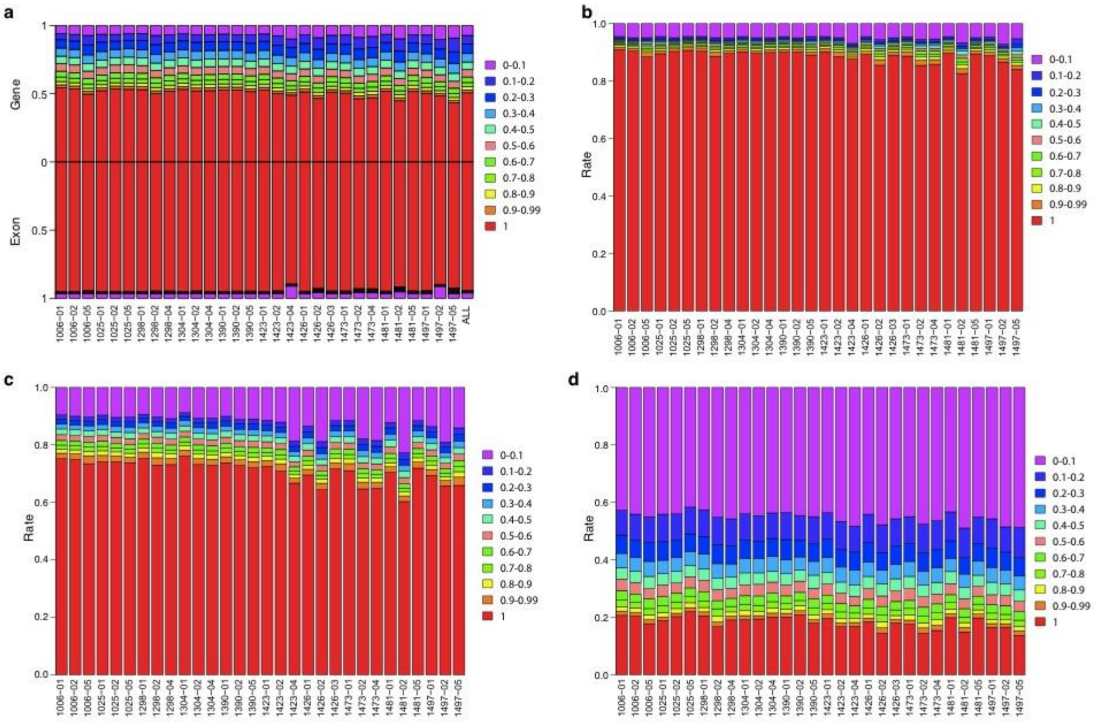

Supplementary Figure 7. Coverage statistics of the *de novo* assemblies (n=30) unambiguous aligned to the reference genome. (a) genes and exons, (b) interspersed repeats, (c) tandem repeats and (d) segmental duplications. Color denotes fraction of genomic elements that are covered by the *de novo* assembly.

Supplementary Figure 8. Mapping of non-ref sequences

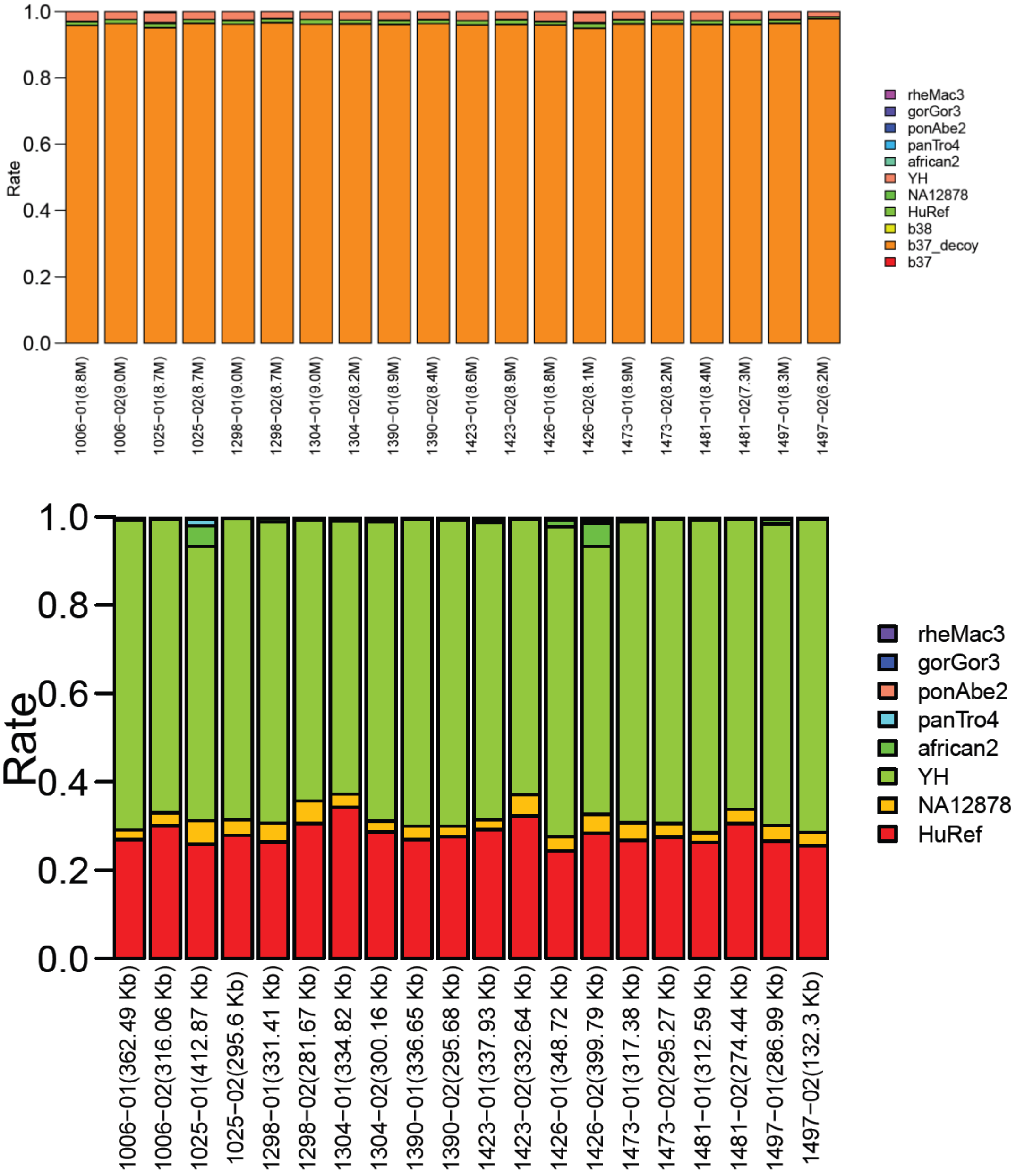

Supplementary Figure 8. Mapping of novel sequences identified in the parents (n=20) against other human and primate genome sequence. x-axis: Total length of the novel sequences (>=100bp) in the individual *de novo* assemblies that are not present in the NCBI b37 (upper panel) and NCBI b37 plus decoy sequence (lower panel); y-axis: Fraction of the novel sequences that are unambiguously aligned to different human and primate DNA resources with > 95% identity and 95% aligned ratio. If one novel sequence can be aligned to multiple resources the priority is: HuRef, NA12878 and YH and african2 over primate sequences: chimpanzee (panTro4), gorilla (gorGor3), orangutan (ponAbe2) and macaque (rheMac3)

Supplementary Figure 9: Genomic distribution of non-ref sequence

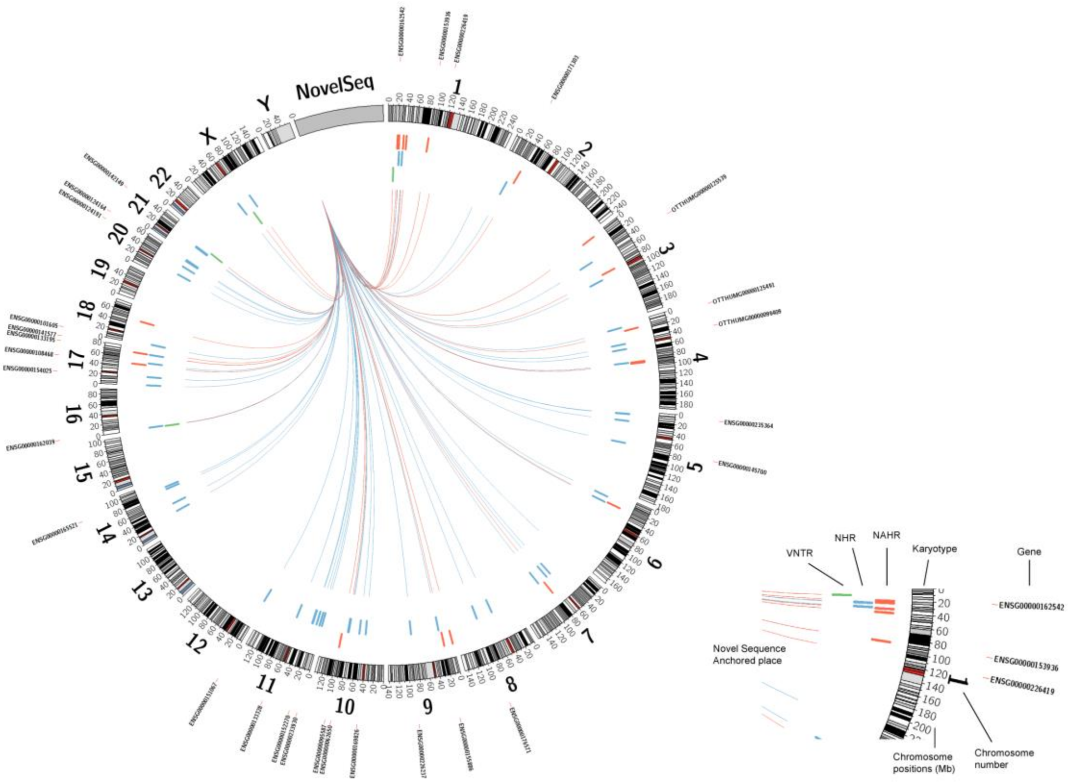

Supplementary Figure 9. The distribution, mechanism, ancestral state, functional annotation of the 1.2M novel sequence that can be localized to NCBI b37.

Supplementary Figure 10. SoapAsmVar pipeline

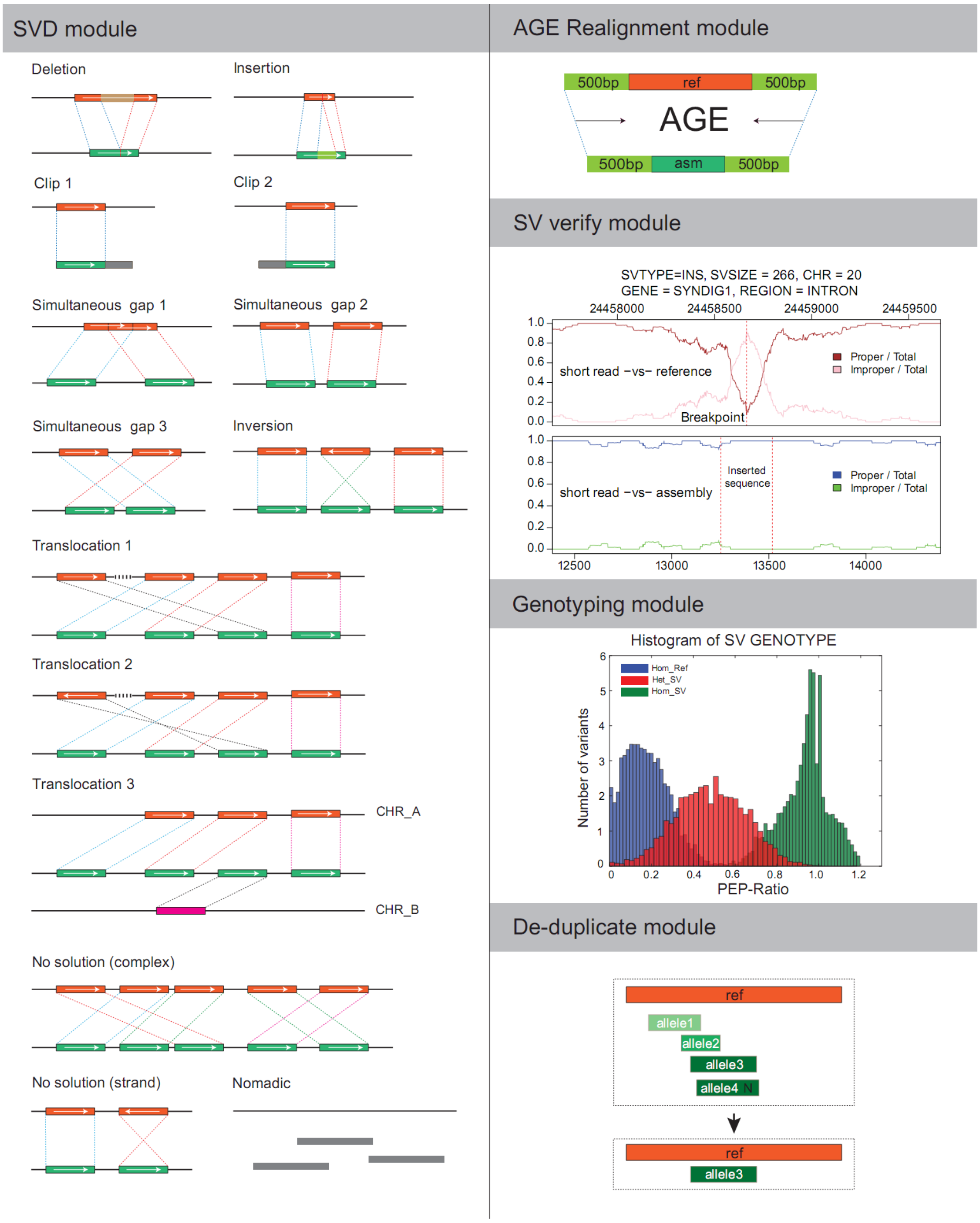

Supplementary Figure 10. SoapAsmVar pipeline to discover and genotype structural variants from *de novo* assemblies. SVD module: A pair of bars connected by dashed line represent an alignment block; Solid black line indicates different alignment blocks; Red bar indicates reference sequence; Green bar indicates assembly sequence which is unambiguously aligned to the reference (misalignment probability <0.01); Gray bar indicates assembly sequence that cannot be unambiguously aligned to the reference. AGE realignment module: Fresh green bar indicates 500bp conservative sequence around the variant breakpoints. SV verified module: Top shows the short-read vs reference

alignment; Bottom shows the short-read vs assembly alignment. Genotype module: A Gaussian mixture model with linear constraints is fitted using the PEP ratio, which is the proportion of proper aligned reads around the variant loci compared with the expected number of proper aligned reads; Solid bar: real data; Dashed line: the Gaussian distribution of the three genotype components in the Gaussian mixture model. De-duplicate module: Intensity of the color represents allele frequency where darker represents higher allele frequency. Whenever there are multiple different non-reference alleles observed within 50bp, the alleles with the highest frequency with lowest gap ratio in the 200bp window around the variant region will be selected. See methods for details.

Supplementary Figure 11: Concordance between GATK and SoapAsmVar

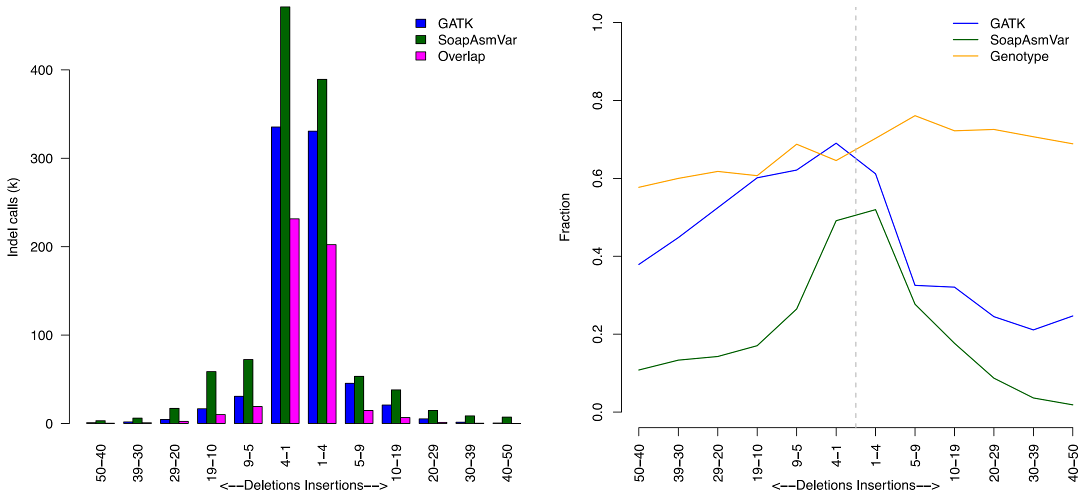

Supplementary Figure 11. Left: Number of indel calls binned by length by GATK haplotype caller (blue), SoapAsmVar (green) and overlapping calls determined using 50% reciprocal overlap (magenta). Average number of calls per individual is 795k and 1140k for GATK and SoapAsmVar, respectively. Right: Fraction of call-set that are also called in the other call-set binned by length. GATK indels also called by SoapAsmVar (blue) and SoapAsmVar indels also called by GATK (green). The concordance in genotype in overlapping calls is shown in orange.

Supplementary Figure 12: Callability given sequencing depth

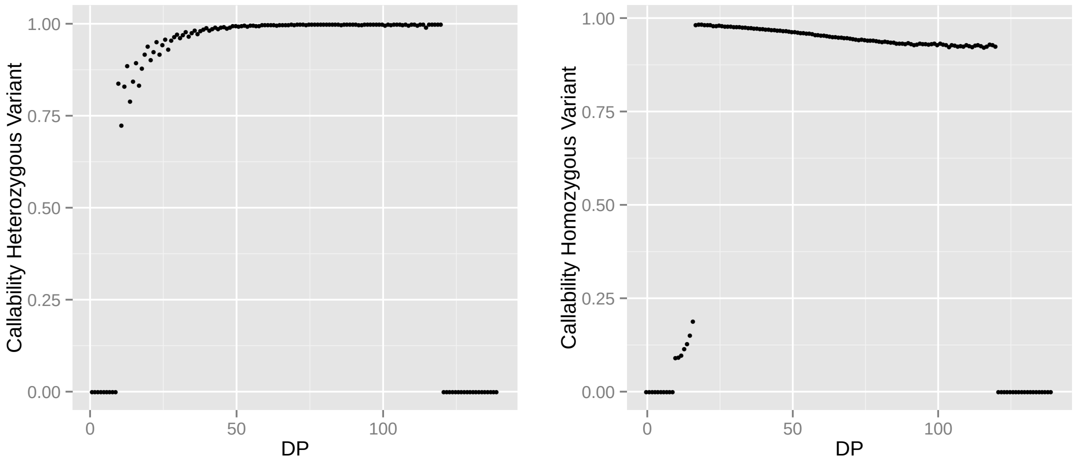

Supplementary Figure 12. The callability (ie. the probability that a true variant passes the conservative quality filters) given the sequencing depth. DP: read depth. The callability of heterozygous variants was calculated based on 3.2 million trio-variant combinations where one parent of the trio was homozygous for the reference allele and the other parent was homozygous for the alternative allele. The callability of homozygous variants was calculated based on 27 million trio-variant combinations where both parents of the trio were homozygous for the reference allele.

Supplementary Figure 13: Determining genotype quality cutoff

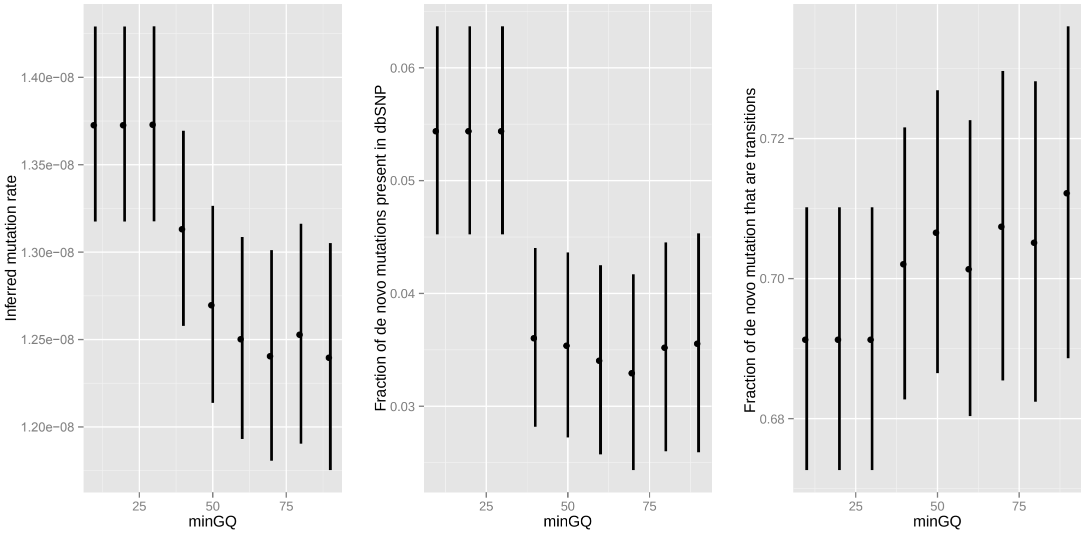

Supplementary Figure 13. The figure shows the effect of changing the minimum GQ (genotype quality) cutoff in the conservative filters used to call *de novo* variants if the other cutoffs are kept constant. We look at three criteria: i) the inferred mutation rate, ii) the fraction of mutations that are in dbSNP and iii) the fraction of mutations that are transitions. All three criteria are stable once the minGQ cutoff is higher than 50. Using the most conservative cutoff (minGQ=90) we find 365 de novo mutations and with the least conservative cutoff (minGQ=10) we find 606 de novo mutations. The error bars represent standard errors.

Supplementary Tables

Supplementary Table 1. Concordance between sequence data SNVs and chip genotyping data.

ID	Overlapping SNPs	Genotyped in both	Concordant Markers	Concordance Rate	Percent Concordance
1006-01	234306	117147	117055	0.999215	99.9
1006-02	234306	116969	116900	0.99941	99.9
1006-05	234306	116966	116909	0.999513	100.0
1025-01	234306	117151	116802	0.997021	99.7
1025-02	234306	116966	116861	0.999102	99.9
1025-05	234306	117151	117055	0.999181	99.9
1298-01	234306	117150	117063	0.999257	99.9
1298-02	234306	116968	116888	0.999316	99.9
1298-04	234306	116968	116897	0.999393	99.9
1304-01	234306	117152	117079	0.999377	99.9
1304-02	234306	116972	116878	0.999196	99.9
1304-04	234306	116969	116907	0.99947	99.9
1390-01	117153	117151	117051	0.999146	99.9
1390-02	234306	116966	116896	0.999402	99.9
1390-05	234306	117152	117052	0.999146	99.9
1423-01	234306	117151	117074	0.999343	99.9
1423-02	234306	116967	116887	0.999316	99.9
1423-04	234306	116962	116834	0.998906	99.9
1426-01	234306	117146	117045	0.999138	99.9
1426-03	234306	116966	116908	0.999504	100.0
1473-01	234306	117150	116933	0.998148	99.8
1473-02	234306	116951	115248	0.985438	98.5
1481-01	234306	117150	116964	0.998412	99.8
1481-05	234306	116977	116475	0.995709	99.6
1497-01	234306	117139	116971	0.998566	99.9
1497-02	234306	116967	116737	0.998034	99.8
1497-05	234306	117149	116969	0.998463	99.8
Total	229967	117055	116864	0.9984	99.8

Supplementary Table 1. Concordance between sequence data SNVs and chip genotyping data. Only 27 individuals were genotyped due to low amounts of DNA in three samples.

Supplementary Table 2. Derived SNV allele frequency

Frequency	SNV Known	SNV Novel
0.025	1,236,966	378,334
0.050	633,903	20,772
0.075	432,092	3,725
0.100	335,243	1,792
0.125	284,648	1,100
0.150	251,159	997
0.175	221,647	568
0.200	196,356	487
0.225	183,297	358
0.250	168,531	312
0.275	159,861	258
0.300	148,649	226
0.325	139,750	166
0.350	131,627	159
0.375	123,614	162
0.400	118,167	154
0.425	113,348	103
0.450	109,086	124
0.475	101,964	99
0.500	99,021	132
0.525	93,076	78
0.550	92,742	63
0.575	87,536	33
0.600	85,453	57
0.625	81,195	57
0.650	78,584	33
0.675	77,438	36
0.700	75,480	36
0.725	74,136	65
0.750	70,525	54
0.775	70,368	54
0.800	68,582	44
0.825	69,056	71
0.850	70,668	209
0.875	71,613	100
0.900	72,091	144
0.925	77,539	180
0.950	84,297	501
0.975	104,976	4,106
Total	6,694,284	415,949

Supplementary Table 2. Derived allele frequency spectrum of non-fixed, bi-allelic SNVs with genotype information in the 20 Danish parents.

Supplementary Table 3. Folded frequency spectrum of indels

Frequency	DEL Known	INS Known	DEL Novel	INS Novel
0.025	69,792	43,740	55,906	44,565
0.050	48,933	32,928	15,400	16,941
0.075	39,675	27,361	10,030	10,968
0.100	33,291	23,659	8,214	8,286
0.125	29,832	21,613	7,098	6,597
0.150	27,263	20,245	6,307	5,539
0.175	25,159	18,550	5,395	4,555
0.200	22,758	17,418	4,501	3,916
0.225	21,731	17,072	3,784	3,313
0.250	20,288	16,163	3,273	2,972
0.275	20,074	15,878	2,787	2,536
0.300	19,010	15,607	2,463	2,226
0.325	18,597	15,262	2,156	2,127
0.350	18,222	15,267	1,903	2,034
0.375	17,753	14,702	1,731	1,796
0.400	17,733	14,982	1,493	1,837
0.425	17,355	14,985	1,217	1,724
0.450	17,274	15,210	1,017	1,681
0.475	16,607	14,908	898	1,596
0.500	8,669	7,370	522	846
Total	510,016	382,920	136,095	126,055

Supplementary Table 3. Folded frequency spectrum of non-fixed, bi-allelic short indels with genotype information in the 20 Danish parents.

Supplementary Table 4. Mendelian error rates

GATK raw calls	SNV	Insertion (<50bp)	Deletion (<50bp)
Total raw calls	9,056,568	872,547	863,053
Sites with Mendelian errors	504,167	293,764	236,221
Sites with (>=2) Mendelian errors	231,180	173,635	127,890
% with at least one Mendelian error	0.06	0.34	0.27
% filtered by Mendelian error constraint	0.03	0.20	0.15

SoapAsmVar raw calls	Insertion (<50bp)	Deletion (<50bp)	VNTR (>=50bp)	NAHR (>=50bp)	NHR (>=50bp)	TEI (>=50bp)
Total raw calls	617,413	776,007	25,363	32,981	22,818	2,901
Sites with Mendelian errors	184,102	227,499	6,432	10,005	7,580	971
Sites with (>=2) Mendelian errors	60,337	74,716	2,051	3,411	2,687	326
% with at least one Mendelian error	0.30	0.29	0.25	0.30	0.33	0.33
% filtered by Mendelian error constraint	0.10	0.10	0.08	0.10	0.12	0.11

Supplementary Table 4. Investigation of Mendelian error rates in raw calls from GATK (upper) and SoapAsmVar (lower) reveals no difference in Mendelian error rates between the two methods. SoapAsmVar sites were filtered if they contained two or more Mendelian errors. NAHR: Non-Allelic Homologous Recombination, NHR: Non-Homologous Recombination, TEI: Transposable Element Insertions, VNTR: Variable Number of Tandem Repeats.
